# The Impacts of COVID-19 on US Maternity Care Practices: A Followup Study

**DOI:** 10.3389/fsoc.2021.655401

**Published:** 2021-05-27

**Authors:** Kim Gutschow, Robbie Davis-Floyd

**Affiliations:** ^1^ Departments of Anthropology and Religion, Williams College, Willliamstown, MA, United States; ^2^ Department of Anthropology, Rice University, Houston, TX, United States

**Keywords:** COVID-19, SARS—CoV—2, maternity care, newborn care, midwives, community births, pregnancy, doulas

## Abstract

This article extends the findings of a rapid response article researched in April 2020 to illustrate how providers’ practices and attitudes toward COVID-19 had shifted in response to better evidence, increased experience, and improved guidance on how SARS-CoV-2 and COVID-19 impacted maternity care in the United States. This article is based on a review of current labor and delivery guidelines in relation to SARS-CoV-2 and COVID-19, and on an email survey of 28 community-based and hospital-based maternity care providers in the United State, who discuss their experiences and clients’ needs in response to a rapidly shifting landscape of maternity care during the COVID-19 pandemic. One-third of our respondents are obstetricians, while the other two-thirds include midwives, doulas, and labor and delivery nurses. We present these providers’ frustrations and coping mechanisms in shifting their practices in relation to COVID-19. The primary lessons learned relate to improved testing and accessing PPE for providers and clients; the need for better integration between community- and hospital-based providers; and changes in restrictive protocols concerning labor support persons, rooming-in with newborns, immediate skin-to-skin contact, and breastfeeding. We conclude by suggesting that the COVID-19 pandemic offers a transformational moment to shift maternity care in the United States toward a more integrated and sustainable model that might improve provider and maternal experiences as well as maternal and newborn outcomes.

## Introduction: Changing Practices and Attitudes Toward COVID-19 Among United States Maternity Care Providers

This article illuminates shifting maternity care practices and protocols among a select group of community- and hospital-based providers across the United States in response to the COVID-19 pandemic during 2020. Following up on an earlier essay (Davis-Floyd et al., 2020) that summarized provider responses about their shifting practices and attitudes early in the COVID-19 pandemic in April of 2020, we expanded our questionnaire and the set of providers we contacted to discuss how attitudes and protocols had further changed in response to new evidence and experience with SARS-CoV-2 and COVID-19 by October and November of 2020. Here, our focus is on the emergent ways in which maternity care providers—obstetricians, midwives, nurses, and doulas—reflect upon their latest adaptations to COVID-19.

Our article illustrates how and why individual providers in both community and hospital settings have shifted their practices and attitudes toward COVID-19 and the SARS-CoV-2 virus. We highlight the still-emergent knowledges and experiences for both providers and childbearers in relation to COVID-19 as well as the critical conclusions drawn during the first year of the pandemic. By the fall of 2020, the climate of fear and loss of control that had dominated the early months of the pandemic had given way to a landscape in which providers had reestablished agency by adjusting protocols to be more evidence-based, while childbearers reestablished some agency by being allowed to bring a birth partner into the labor and delivery process once again. We close by considering how the COVID-19 pandemic offers both a disruptive moment and key lessons for developing more integrated, sustainable, and resilient US maternity care system that can benefit providers, mothers, and newborns.

## Methods

Between September and December 2020, we conducted an email survey of maternity care providers about their practices and attitudes in response to COVID-19. We emailed a questionnaire (see Appendix) to a list of providers we had developed in the spring of 2020 while researching a rapid response article on COVID-19 and maternity care (Davis-Floyd et al., 2020) and used snowball sampling to enable our respondents to forward our questionnaire to other providers. We also posted our survey questions on the REPRONETWORK listserv. All of our respondents replied to our survey questions *via* email, while some briefly discussed their responses with us or replied to all providers on our list, thereby enabling those who responded later to have the benefit of prior responses. Given the constraints of a raging pandemic, most providers responded briefly. All respondents gave explicit written consent for their comments to be used, and most indicated that they wished to be identified, while a few preferred to remain anonymous.^1^ Unless otherwise indicated, real names will be used for our respondents.

By November of 2020, 28 providers had responded to our survey, roughly one-third of whom were obstetricians (one was also a maternal-fetal medicine specialist), and two-thirds of whom were midwives (certified professional midwives—CPMs—and certified nurse midwives—CNMs), doulas, and a labor and delivery nurse. These providers came from Texas, Arizona, Arkansas, Virginia, North Carolina, Florida, Illinois, Idaho, Oregon, Massachusetts, and California.

We frame the provider responses within a summary of the most recent labor and delivery and newborn care guidance in response to COVID-19 and SARS-CoV-2 in the United States as of December 2020. Our literature review of this guidance drew on a repository on “COVID-19, Maternal and Child Health, and Nutrition” compiled monthly by the Johns Hopkins Center for Humanitarian Health, and keyword searches for critical terms.^2^ We emphasize that evidence and provider experiences are rapidly evolving, and many systematic studies of the impacts of SARS-CoV-2 and COVID-19 on pregnancy, maternal health, and neonatal health remain to be conducted, while case studies offer only limited evidence. The views and findings described herein should be considered as provisional responses to an evolving pandemic. Our article is organized around the salient themes that emerged from our data and our questionnaire.

## Findings: Shifting Attitudes and Practices

### Shifting Attitudes Toward Covid-19: From Fear to Action

Looking back with the hindsight of current knowledge, we cannot stress enough how disruptive and confusing the evidence around SARS-CoV-2 and COVID-19 was in the first half of 2020. The prior coronavirus epidemics of SARS and MERS, as well as experiences with Ebola and Zika viruses, compounded the trepidation around rates and routes of transmission of SARS-CoV-2, case fatality rates for COVID-19, and its specific impacts on pregnant women. Providers were responding to patient fears and misinformation, as well as to a fundamental lack of evidence with regard to how SARS-CoV-2 and COVID-19 would impact pregnancy, maternal, and newborn outcomes. In this article, we capture the shifts in their attitudes and practices from spring to fall 2020.

During the winter and spring of 2020–21, COVID-19 continued to spread rapidly and widely across the globe, often *via* asymptomatic transmission, and many regions had faced second and third waves of contagion that were even more virulent than the first. By the end of May of 2021, there were over 167 million cases and over 3.5 million deaths globally, as well as over 33 million cases and nearly 600,000 thousand deaths in the United Stated alone.^3^ While the United Stated only accounts for 4.25% of the global population, it was responsible for roughly one-sixth of the global death count. With over 140 million births expected worldwide in 2020, and many pregnant women at risk for being infected with SARS-CoV-2, hospitals and institutional bodies rushed to establish protocols and guidance for labor, delivery, and postpartum care (Boelig et al., 2020a,2020b; Stephenson et al., 2020).

Providers’ initial fears about maternal and newborn outcomes were not surprising, given the high rates of obstetric complications that pregnant women had faced during other recent coronavirus outbreaks, namely SARS and MERS (Schwartz and Dhaliwal 2020), as well as during Ebola and Zika (Strong and Schwartz 2019). While the SARS outbreak had a global case fatality rate of 11%, maternal mortality was as high as 30%, with 60% of pregnant women requiring admission to an ICU and 40% requiring intubation (Schwartz and Graham, 2020). A systematic review and meta-analysis of 19 studies analyzing pregnancy outcomes for women with confirmed SARS, MERS, or COVID-19 infections reported significantly increased rates of obstetric complications than for women without coronavirus infections (Di Mascio et al., 2020).

Rates of obstetric complication from COVID-19 were alarming (Boelig et al., 2020a), and significant increases in maternal death, stillbirths, and rates of postnatal depression were reported (Ellington et al., 2020; Chmielewska et al., 2021). For a small number of pregnant women, the most serious complications of COVID-19 included severe pneumonia, cardiomyopathy, thrombosis, and multiorgan diseases that require intensive care and mechanical ventilation (Schwartz et al., 2020b). The pooled meta-analysis of several early studies indicated the following rates of maternal and newborn complications: 41% preterm delivery before 37 weeks, 15% preterm delivery before 34 weeks, 15% preeclampsia, 19% premature rupture of membranes (PROM), 91% cesarean delivery, 7% perinatal death, 43% fetal distress, and 9% of newborns admitted to a NICU (Di Mascio et al., 2020). While miscarriages and stillbirth were rare, women who were asymptomatic during labor and delivery fared much better, and some preterm births were provider induced (Boelig et al., 2020a).

As 2020 progressed, the landscape of maternity care shifted as providers realized the significance of asymptomatic community spread and the dangers the virus posed to mother and newborns. It was estimated that 25–40% of transmissions occur before onset of symptoms and asymptomatic infections can range from 20–50% within select studies (Meyerowitz et al., 2020). The degree and rate of vertical transmission between mother and newborn, in three possible ways—intrauterine or placental transmission, intrapartum transmission, or postpartum transmission—is still being quantified, although early studies indicated that many infected newborns were asymptomatic or developed only mild cases of COVID-19 (Schwartz et al., 2020b).

Our survey respondents recognized that their clients’ fears ranged widely; as obstetrician George Walters (a pseudonym), described: “We are impressed with the wide range of patient perceptions. Some remain near emotional paralysis due to fear and others are not worried at all.” The most common fears about COVID-19 that our providers encountered among their clients were:

• Fear of being infected by SARS-CoV-2 or developing COVID-19
• Fear of being denied a labor support person
• Fear of having to choose between a partner and doula for labor support
• Fear of transmitting the virus to a newborn
• Fear of being separated from their newborn after delivery
• Fear of isolation during pregnancy, labor, and the postpartum period

We address all of these concerns herein, as we show how provider attitudes and practices adapted within the following areas:

• Shifting norms for PPE (personal protective equipment) and testing of both providers and childbearers
• Shifting norms about allowing support persons during labor, delivery, and the postpartum period
• Shifting norms about separating mother and newborn vs. “rooming in” in response to the shifting evidence of vertical transmission of SARS-CoV-2 from mother to newborn
• Ongoing lack of integration between community- and hospital-based providers

Our findings suggest that the changes in provider knowledge and protocols have had significant impacts on women’s mental health, with downstream effects on newborn and maternal health that remain to be quantified but are just beginning to emerge. Homebirth obstetrician Stuart Fischbein of Los Angeles summarized how the hospitals in his area used COVID-19 as a pretext to abandon their “mother-baby friendly” practices in ways that compromised maternal health and agency:

The pandemic has exposed the medical model of maternity care and clarified how they really think. The Mother-Baby Friendly moniker that they were all so proud of labeling themselves went out the window immediately. Little or no concern for the psychological well-being of the mother is clear by their separation policies…For that matter, the pandemic has exposed the fallibility of experts and trust in their judgement which I think is a good thing. The individualization of care and respect for autonomy in decision making should not go out the window because of fear.

Fischbein’s comment speaks to the relatively recent humanistic changes in many United States hospitals that are termed “Mother-Baby Friendly” because they allow partners and doulas into the labor room, immediate skin-to-skin contact after birth, and newborn rooming-in. He notes how quickly these humanistic policies of connection were reversed in favor of the traditional medical model of obstetrics, which separates mothers and newborns as well as mothers from their families.

Doula Stevie Merino, cofounder of Doulas of Color Training and Birthworkers of Color Collective in California, summarizes how her experiences have improved her protocols:

People, including providers and clients, are all operating and approaching this time with different emotions, fears, anxieties, and beliefs. I have found that there have been major bumps in the road as we are all navigating this unprecedented time…now that I have tested experience through this pandemic [I] feel more confident about best practices/protocols.

CNM Dinah Waranch, who attends birth at home and at a birth center in Texas, notes that: “Pregnant women are by definition somewhat anxious. Now some of them are more so. On the other hand, just like the country, the clientele is divided.” CNM Jenny Bagg, who practices at a community health center in Florida, reflects on the risk of catching COVID-19 at her center vs. the community: “Where I am, the community is a much more likely place to catch COVID than in the hospital. Patients generally do not seem afraid of contracting COVID in the hospital.” Speaking to both women’s and providers’ fears, obstetrician George Walters said:

Many feared early on. But now that we know the rate of infection is low, patients are much less concerned about going to the hospital. I would not say we practitioners are afraid of catching COVID. Our entire careers have involved infection risk. We adapt and move forward. We don’t like wearing masks, but we believe they help.

The notion that providers and clients no longer fear COVID-19 may not be widespread; for example, doula Stevie Merino expressed a rather different view:

Pregnant people definitely are reporting not feeling safe and more at risk of contracting COVID-19 at hospitals which is why so many are inquiring about other options. I personally am afraid of contracting COVID-19—as a self-employed person and as a single parent, I do not have the same benefits and protections that others have during this time.

Homebirth obstetrician Fishbein explains that his clients’ primary fear is not their health but hospital policies that may be driving them to pursue the “other option” of community birth that Merino alludes to; Fishbein says, “Main fear is not about health. It is about hospital restrictive policies and separation from their baby and support system.” Doula Merino echoes these fears about hospitals:

Not being able to have the support that they want because of hospital support person restrictions is the number one fear that I have heard the most. Another is the limited support options because of the fear, anxieties, risks of COVID-19 for themselves/infant(s).

Obstetrician George Walters explained why fears of COVID-19 were high but later abated:

We are very thankful that reproductive age women are mostly unaffected by COVID. We were initially worried that it would be worse than H1N1. And we are even more grateful that newborns appear to be almost fully unaffected.

MFM specialist Charles Deena (a pseudonym) summarized the initial confusion around the major safety protocols being instituted at his large urban hospital in Illinois:

As for safety protocols, a lot of this had to do with where COVID-positive people were allowed to labor (on L&D? In a separate unit?), how to deal with particular emergencies in a COVID+ patient (i.e., maternal code, need for intubation, need for emergency cesarean delivery or operative vaginal delivery), and contingency planning for patients who needed advanced life support (i.e., intubated on ventilator, need for ECMO).

L&D nurse Hicks from Texas explained how client fears were reduced by shifting hospital protocols around PPE and testing:

The mothers I have worked with have expressed a generalized fear of contracting the virus in the hospital. For a lot of them, going to the hospital is one of the first times they have left their homes outside of OBGYN visits…The precautions taken by the facility seem to ease the fears pregnant mothers and their families have.

This range of attitudes toward COVID-19 shows both overlap and differences between community- and hospital-based providers. Overall, we found that hospital-based providers had better access to and control over PPE, testing, and restrictions on their clients than community-based providers. Many of our providers reported that the most significant changes in their protocols involved strict use of PPE, hygiene, testing, telehealth, and restrictions on support people and rooming in, which we discuss in turn below.

Labor and delivery guidance established in the United States by May 2020 included: encouraging oxytocin use at higher doses to shorten duration of labor; using amniotomies for dysfunctional or delayed labor; using prophylactic oxytocin during the third stage of labor to prevent hemorrhages; using early epidurals to minimize need for general anesthesia (which risks aerosolization of the virus); limiting the second stage of labor; performing cesareans if labor had arrested after only 4 h; limiting antenatal corticosteroids after 34 weeks; judicious use of magnesium sulfate for slowing preterm labor because it can cause respiratory suppression; avoiding aggressive fluid hydration; and limiting frequency of cervical exams (Boelig et al., 2020a; Stephenson et al., 2020). Many of these recommendations had little evidence base and overturned years of evidence in support of the more humanistic, holistic, and patient-centered care that birth activists had long fought for (Gutschow et al., 2021; Davis-Floyd, 2018).

Hasty guidance that lacked significant evidence included encouraging cesarean delivery for women who tested positive––estimated at 70% globally by April 2020 according to one systematic review (Debrandere et al., 2020); encouraging inductions and instrumental delivery; isolating newborns from mothers who tested positive; and not delaying cord clamping (Favre et al., 2020). Much of this guidance lacked evidence (Schmid et al., 2020) and some even promoted obstetric violence (Sadler et al., 2020).

The major protocols to protect risk of transmission that were reported in the literature by May 2020 included: universal PPE for providers, childbearers, and support people; universal testing of providers and pregnant women admitted to facilities; limitation to one support person for the entire labor, delivery, and postpartum period; preference for virtual labor support if possible; and no children younger than 16 permitted at any time (Boelig et al., 2020a). For out-patient visits and pregnancy consults, major recommendations included universal PPE for providers and pregnant persons; universal testing and screening before any in-patient visit; postponing elective visits if possible; telehealth for most routine prenatal consults; and keeping additional providers at home if possible (Boelig et al., 2020b). We discuss these shifting protocols and practices in turn below.

### Shifting Practices: Using PPE and Incessant Sanitizing

Our first article indicated that both hospital- and community-based providers suffered severe shortages of PPE in March and April of 2020. By October of 2020, many of the PPE shortages had been resolved, although the fall wave of COVID-19 brought increased stress to hospitals and communities that had not experienced a first wave of COVID. Obstetrician Walters described the shift at his urban hospital in North Carolina:

We were initially short of PPE. But we live in a great community. I worked for about 3 weeks in masks donated by a nail salon. A local distillery (that usually makes alcohol to drink) started cranking out hand sanitizer. You have to love American ingenuity!! We have had no supply problems in months.

Obstetrician Marco Giannotti of Texas representatively reported: “Our office staff all wear standard medical masks. Cloth masks not allowed for staff, and we provide free masks for anyone who needs one (patients and employees),” while MFM specialist Charles Deena reported that by September, his Illinois hospital had “sufficient PPE at this time.” L&D nurse Hicks explained how her hospital is ensuring a steady supply of PPE for all staff:

The major changes in the practices and protocols at the hospital I work at are geared towards protecting patients and healthcare staff from each other…. Our facility has enough PPE but is taking precautions to not run out. Every nurse in the emergency room and labor and delivery unit has to wear an N95 at all times, goggles during patient interactions, and face shields during deliveries, because there is always a chance that a patient will come to the unit that needs emergent care and is COVID-19 positive. All other nurses wear surgical masks at all times. The only time masks can be removed is in the designated break room. The nurses that have to wear N95 masks wear their mask until a string breaks or it gets dirty, which normally take 2 days.

CNM Bagg confirmed that her community health clinic “is doing an excellent job at protecting us as much as possible. We all have multiple N95 masks which are required to be worn at all times in the clinic.” LM (licensed midwife) Jessica Willoughby, who runs a birth center in Florida, described an initial difficulty in getting masks that later resolved:

[Now] we offer surgical masks if people do not come in with masks. We are requiring masks. We are not limiting people at visits or at births. [After initial delays] we have adequate masks….K95s and surgical masks.

Doula Stevie Merino, who makes home visits and attends hospital births, described her difficulty with accessing PPE in California:

It has been difficult for me to access sufficient PPE because I am not a medical provider. Many of the sites that I normally would purchase from are directing them understandably to medical providers/locations. Thankfully, many in my community have been great at supporting with PPE…. I also use continuous PPE gear when visiting homes or at births, even when clients and others have become more relaxed with it.

Community-based midwife CNM Dinah Waranch reported that at her home and birth center practice, “During labor and birth the mother is NOT masked… Midwives are masked. Support people … masked …. Some (clients) roll their eyes at masks.” Waranch told a story about an intake visit that expresses both her own flexibility and that of the midwifery model of care concerning a client for whom she had previously served as a midwife:

[My client] is unmasked and I make a gesture across my face for her to mask up as I am. [She] rolls her eyes, puts on a mask, and stomps into the room. “Masks are communist. They are un-American.” Loudly through her mask, defiant. I am opinionated too. “Communism isn’t so evil,” I am smiling, teasing, but my dagger glints. Then reaching deeply for my mature, inner midwife, I say. “If you prefer not to wear a mask, let’s sit outside in the park a few feet away from each other. I can do your intake history on my phone.” How easy it was then to create a peace between us, to open to each other across the picnic table beside the pond; the story of her motherhood, unique but mutually understood. Our angers soften...

CPM and DO (Doctor of Osteopathy) Sarita Bennett, who runs a midwifery practice in Virginia, explained that her staff midwives “do not wear masks during the birth … all of our birth team members are relatively “non-social” on a good day, and do not frequent some of the higher risk areas like bars, churches or indoor group events.” Homebirth obstetrician Fischbein noted:

Science is compromised. Healthy people have little to fear. Compromised and elderly people should take precautions. We have made no major changes in our practice. We wear PPE at client request. Otherwise, my team and I are choosing to believe much of the suppressed literature that many of the recommended precautions are not evidenced based. We have a trust in our immune systems...

Fischbein alludes to a holistic belief that immune systems are critical to understanding human physiology as well as the physiology of birth. His view also reflects a broader critique of medicalized obstetrics that we have explored elsewhere (Gutschow et al., 2021). Scientists who study viruses still have many questions about why some populations or individuals are less impacted by SARS-CoV-2 and COVID-19 than others (Mukherjee, 2021; Zimmer 2021), as well as about how the evolutionary processes that created birth physiology intersect with those that produced human immunity to viruses and other pathogens.

Besides PPE, many providers reported a strong emphasis on hygiene and sanitation. Echoing other midwives, CPM Sarita Bennett instituted “a short break between clients to allow time to wipe things down and ask that children not come along to prenatal because we can’t wipe down toys every time.” Echoing what other community-based midwives stated, LM Willoughby reported:

Cleaning, everything, all the time, between every patient. It. Is. Exhausting. We also have a hand sanitizer and an alcohol wipe station at the front door…We give isopropol alcohol wipes to the patients when they arrive to wipe down their phones. We as a staff make it a habit to wipe down our phones with those wipes several times a day and hand sanitize frequently...It was a crash course in PPE and I’m so glad I was able to connect with other birth center owners to go in on masks purchases because initially we were unable to find anything!!

She continues by placing extra stress on the drawbacks of these incessant sanitizing efforts:

I always felt like the birth center was a sanctuary from the craziness that happens in the mainstream medical model. Now with COVID, I feel like our tranquil borders have been breached! I hate the super vigilance and paranoia I feel with the obsessive cleaning.

MFM specialist Charles Deena confirmed the benefit of increased PPE use: “Thus far, with our sufficient PPE, only a minority of clinicians have acquired COVID-19. We, luckily, have not had any colleagues die from COVID-19 exposure.” By November, none of the providers who responded to our questionnaire had contracted COVID and none reported any colleagues who had died of COVID-19, although several had to self-quarantine or to quarantine staff. L&D nurse Lauren Hicks described the careful quarantine and contact tracing protocols her hospital conducted:

Luckily, we have only had one nurse test positive. Unfortunately, she worked on the unit before she knew she was positive and had a patient that required rapid response, meaning nearly every staff member on the unit was in the room with her to help her patient. Everyone was wearing masks, so luckily no other staff member became sick. The COVID-19 positive nurse quarantined for over two weeks until her symptoms were gone. Every person that was in contact with her during the shift she worked was contacted and asked to record their symptoms for 2 weeks. We were told if symptoms started to present to contact the unit director and go to the hospital to get tested.

### Shifting Practices: Testing Providers for COVID-19

In contrast to our first survey (Davis-Floyd et al., 2020), in which many providers reported that they had to beg or plead for testing, by November of 2020, there was improved access to testing for many hospital-based providers, but less for community-based providers. The early months of the pandemic had revealed the unpreparedness of United States healthcare and maternity care institutions for a pandemic. Yet many hospitals began to acquire testing capacity and to require testing for all admitted pregnant women as well as regular testing of staff. However, according to an obstetrician at a large teaching hospital in Massachusetts, the demand for testing so outstripped supply that weekly testing was initially required only for teaching staff but not for clinical staff or support staff. In contrast, CNM Jennifer Bagg, who works at a health center in Florida, reported, “The entire staff (over 200 people) are tested every other Monday.” Doula Stevie Merino made a personal choice to get tested: “As a birthworker, I have made the individual choice to be tested every few weeks or more if between births. Los Angeles and Long Beach have free testing sites which I have used and found very efficient, useful.”

Midwife Jessica Willoughby of VIrginia elaborated:

We do not test our patients and staff at the birth center. Every staff member who has been sick I’ve made them go to the urgent care…to get tested…they’ve been really good to us to get our results back quick. I was sick this week and I went in and was given a rapid screen (negative) and a PCR which came back in 48 h (also negative). I have zero tolerance for people being sick. If my staff are sick, they cannot come into the birth center for 14 days unless they have a negative COVID screen.

Willoughby illustrates the value of giving all birth providers access to regular testing with rapid results in communities as well as hospitals. The irregular access to testing and frequent delays in test results for much of 2020 across the United States represent a lost opportunity. By getting tested regularly and having their results rapidly available, providers could limit inadvertent transmission, reduce fear among clients, and limit their own anxiety about attending asymptomatic clients. MFM specialist Charles Deena, who works in an urban hospital in Illinois that handles 12,000 births a year, alluded to ongoing difficulties in accessing tests for some providers: “(Testing) is a point of contention, especially given the needed resources to do universal testing. Test positive rates are pretty varied across landscapes, with the highest being in NYC, though our test positive rate…is (also) relatively high.”

### Testing in Community vs. Hospital Settings

Some of our community birth providers required their pregnant clients to be tested, while others did not. Community-based CPM Shea Childs in Arkansas notes that she would not test asymptomatic clients, but feels differently about symptomatic clients, “If they were symptomatic at 36 weeks or more, I may (test), but that has not come up.” Community-based midwife Jessica Willoughby requires her staff but not her clients to get tested regularly: “We do not require COVID testing. I’ve never even sent a mom to get tested. If she’s asymptomatic we just treat everything as normal. If she’s sick we ask that she stay home, and we can do telehealth. I’ve never had a patient be sick in labor.” Many community-based providers work with a clientele who are low risk for birth complications as well as COVID-19, and who follow social distancing and masking guidelines.

In contrast, our hospital-based providers were very serious about mandatory testing, reporting that all childbearers are routinely tested before admission to hospital for labor as well as for out-patient pregnancy consults. Yet there were difficulties, as CNM Jennifer Bagg of Florida explained: “We have started testing all pregnant patients for COVID weekly starting at 36 weeks. We do the rapid antigen tests but the whole process from start to finish takes over 30 min and severely negatively impacts our already very busy patient flow.”

Hospital-based CNM Kylea Liese of Chicago said that patients in her hospital are “tested a few days prior if they have a scheduled c/s or induction.” About office visits, obstetrician George Walters stated, “We prescreen with questions about symptoms and contacts every person prior to entering the building. Patients wait in their cars, not the waiting room.” Texas-based obstetrician Marco Giannotti confirmed the prevalence of out-patient testing: “The biggest change has really been in screening patients before entering the office.” Regarding office visits for mothers who test positive for the virus, CNM Bagg spoke representatively: “We try to make them the last appointment of the day to limit others’ exposure, don full PPE and try to finish the visit as quickly as possible.”

Community-based CPM/DO Sarita Bennett of Virginia described the scarcity and unreliability of testing:

Testing has been difficult to access, unreliable in its results––we have seen some negative results that we didn’t believe and treated the person as positive and been exposed to people who a week later told us they had had an asymptomatic positive test which resulted in quarantines but no further sickness. Many of our clients have no insurance or have difficulty accessing testing. Most, if not all, have protected themselves through staying out of public, masking, hand washing, etc. The testing seems the least effective way of screening at this point.

Community-based CPM Debbie Query of Arkansas adds:

I have read the remarks from the scientist who developed the test, which is not … considered reliable. Nor am I any more concerned about this virus than any other virus. I have always been cautious about germs and cleanliness and so my practice has pretty much stayed the same. The only change is if they or somebody in the family tests positive I will postpone their prenatal or do a “tele-med” call.

MFM specialist Deena from Illinois elaborated:

interesting to note the differences in testing, especially the weekly testing (which seems aggressive to me) and the use of different testing platforms (i.e., rapid antigen versus PCR testing)….We will screen people with nasopharyngeal PCR swabs upon admission as we have a test that will produce results pretty rapidly.

These providers were alluding to the main diagnostic test for COVID-19 used in 2020, the Reverse Transcription Polymerase Chain Reaction (RT-PCR) test. This test initially extracts viral RNA from the sample, uses an enzyme to convert viral RNA into DNA, and subsequently passes through several steps to amplify viral RNA with multiple cycles. The sensitivity of this method is so great that even non-infectious fragments of viral nucleic acid can yield positive results for an asymptomatic individual (Surkova et al., 2020; Kaufman and Puopolo, 2021). We emphasize that testing positive indicates that an individual is infected with the SARS-CoV-2 virus, although such individuals are frequently labeled as “COVID-19 positive” in ordinary discourse. By February 2021, much of the medical literature we consulted distinguished between SARS-CoV-2 and COVID-19; we follow that distinction here.

A systematic review and meta-analysis of 30 population-based studies conducted in September 2020 revealed that 95% of all obstetric patients were asymptomatic (Yanes-Lane et al., 2020). In the future, careful distinctions may be made between asymptomatic but infected individuals and the smaller number of infected persons with symptomatic COVID-19. This distinction mirrors the critical distinction between being HIV positive and having a diagnosis of AIDS.

Our providers indicated a rise in telehealth, especially for doulas who reported attending to their clients virtually, using devices positioned in the sight line of the laboring person. Given that providers in the room could shut off or move the device out of range without consent of the laboring person, and that the essence of doula care is physical touch and presence, many doulas were unsatisfied with virtual support. In contrast, many providers in both hospitals and communities were comfortable using telemedicine for prenatal care. While homebirth midwives have mixed opinions about telehealth, hospital-based providers were more comfortable with this form of care. Obstetrician Lucia Gomez (pseudonym) from Texas confirmed that, “Our offices had telemedicine appts for both gyn and OB patients,” yet obstetrician Marco Giannotti reported that his practice never went to telehealth. Obstetrician Marilyn Vanover had a negative opinion about telehealth, stating,

My biggest concern is the decrease in in-person visits to assess patient needs. I am also concerned that this will become the “norm” too often once the pandemic is under control. I am concerned about the delay in diagnosis of ectopics and PID [pelvic inflammatory disease].

We will need longer-term and more systematic studies to determine whether the rise of telemedicine in maternity care continues after the pandemic passes.

### Restricting Labor Support: Impacts on Maternal Mental Health and Health Equity

By mid-March and into the summer of 2020, many hospitals across the United States had begun to exclude labor support people—partners and doulas—due to fears of COVID-19 transmission (Davis-Floyd et al., 2020). In the United States, an Executive Order by New York State Governor Cuomo on March 28, 2020 explicitly allowed one support person to attend the person in labor, and other hospitals later adapted their policies around labor support people.^4^ By October 2020, most of our respondents reported that their hospital or clinic allowed at least one support person, and sometimes even for women who tested positive.

CNM Diana Jolles from Arizona stated that her hospital began excluding all support people in April of 2020 but reallowed them back in September 2020. Obstetrician Walters echoed other hospital-based providers when he said, “We never excluded a support person. Our unit continues to allow one support person. That is usually the father, but other times a family member. Rarely a paid doula.” Even birth centers were limiting support people, as confirmed by several of our provider respondents. CNM Dinah Waranch described more flexible limits at her birth center in Texas: “One support person only encouraged but additional support people at mother’s discretion, masked. Family arriving after birth not encouraged.”

Obstetrician Michael White of Texas described how the limitations on support people restricted family access during prenatal care, labor, and delivery: “We no longer allow any other family to accompany them, thus spouse and family are excluded from the prenatal care. At the hospital level it too has severely restricted family access to a delivery.” Several providers reported that the situation had become quite difficult for doulas; doula Roselyn Faith from Oregon explained that:

The local hospitals stopped allowing doula support for birthing mothers from March until now [September 2020]. They are just now opening their policies a bit, yet only for paid doulas. The volunteer program I was participating in still isn’t allowed. This was a program offering birth support to all women and serving mostly low income and women of color. I’m hoping this program will be continued very soon.

Doula Stevie Merino explained how confusing these rules were, as well as how the limitations on support people put her clients and doulas in difficult situations:

Every hospital’s policies are different which has also been difficult to navigate and keep up with…There are very few hospitals that see doulas as an essential part of the birth team, which has allowed me and partner/support person to be present in the room. In quite a few instances, I have been chosen over a partner to be present in labor. This was an intentional and very difficult decision on all parties.

By not considering doulas as “essential personnel,” hospital protocols devalued their services and limited the ability of childbearers to advocate for themselves and their newborns (Searcy and Castañeda, 2020). Even when hospitals allowed a single support person, the strict rules insisting that this support person was forbidden to leave the labor room further limited or prevented continuous support in labor, as some families cannot afford for the partner to stay the entire time. This rule can fall especially heavily on minoritized and low-income childbearers, who have been struck hardest by the virus (Obinna, 2020; Norton et al., 2020). Further, it penalizes women who already had small children at home with limited childcare, as their partners might have to choose between tending to their children or their birthing partner, who is facing increased stress and isolation (Norton et al., 2020; Almeida et al., 2020).

Speaking of his teaching hospital, MFM specialist Charles Deena described that for childbearers who tested negative, or who tested positive but were asymptomatic:

one support person is allowed with them. Doulas are allowed, but they count as the one support person. From my experience, most folks choose their partner. As for postpartum, if one is COVID-19 negative, you can have up to 2 people visit, although 1 can stay overnight.

Doula Merino described the lingering effects of the isolation that childbearers faced:

Many are not having the experiences that they envisioned in terms of family, friend, community support due to social distancing recommendations. This isolation has had and will continue to have dramatic effects on postpartum people and new parents.

The restrictions denying labor support for childbearers who tested positive could indeed mean isolation and mental suffering, as MFM specialist Deena described:

COVID-19 positive pregnant people who labor in our hospital do so in a negative pressure room on a floor above labor and delivery, have one-to-one nursing, and only one provider (no residents) at the delivery. As for the experiences of people laboring alone…the stories I heard from my colleagues working on the [COVID] floor is that it was heartbreaking––extremely isolating and really difficult to help people through, especially since we knew (and still know) so little about perinatal outcomes associated with the virus.

L&D nurse Hicks described the alienating scene that mothers testing positive faced:

COVID-19 positive women…were not allowed to have a support person with them, and the newborn was immediately removed after delivery. Full PPE is worn while in the negative pressure room, which includes a N95, googles, face shield, gown, hair cover, and shoe covers. Nurses are encouraged to cluster care while in the patient’s room. When nurses are caring for COVID positive patients, a primary nurse is allowed to go into the room while another nurse acts as a runner to get any supplies or medications the primary nurse needs.

This denial of labor support is especially critical for women of color, who have been disproportionately affected by COVID-19 and who already face formidable disparities in maternity care and obstetric outcomes (Ellington et al., 2020; Norton et al., 2020; Obinna 2020). Well before COVID-19 struck, between 2014 and 2017, the pregnancy-related mortality for non-Hispanic Black women (41 deaths/100,000 live births) was three times that of non-Hispanic white women (13.4 deaths/100,000 live births) and quadruple that of Hispanic or Latina women (11.6 deaths/100,000 live births) [Centers for Disease Control and Prevention (CDC), 2020a]. Evidence shows that the racial disparities in maternal outcomes are related to the chronic stress of structural racism as well as providers’ racial bias (Bridges 2011; Eichelberger et al., 2016; Davis 2019; Valdez and Deomampo, 2019; Obinna 2020).

By defining doulas as visitors, not essential personnel, childbearers are being denied critical advocates during labor and the postpartum period when they are isolated due to COVID restrictions. A Cochrane meta-analysis of deliveries in 17 countries found that women receiving continuous labor support had shorter labors, were *more likely* to have spontaneous vaginal delivery and report positive childbirth experience, and *less likely* to have a cesarean delivery, to use any form of intrapartum analgesia, to have a baby with low (<5) Apgar score, and to have postpartum depression (Bohren et al., 2017). Yet a Canadian study (Fortier and Godwin, 2015) showed that doula presence was not viewed favorably by half of the obstetricians and one fourth of nurses in the study. Given this level of hostility to doulas, we are not surprised that the COVID-19 pandemic provided quick justification to exclude them from labor and delivery rooms, with adverse consequences for women that remain to be fully quantified.

There is evidence that laboring alone without support while sick with COVID-19 can have negative impacts on both mothers and newborns. One systematic study of 2,417 women from Massachussetts General Hospital, which compared women testing positive for SARS-CoV-2 with matched controls, found that women testing positive reported higher levels of pain during delivery, lower newborn weights, more newborn admissions to a NICU, and were 11 times more likely to have no visitors during labor and delivery (Mayopoulous et al., 2020). Further, many of these adverse effects were explicitly associated with the absence of labor support persons, proving that isolation itself (not just being seropositive) has detrimental maternal effects. Nearly half of the women who tested positive reported clinically significant acute stress symptoms (Mayopoulous et al., 2020). A Canadian study showed that after the onset of COVID-19, 37% of women had elevated depression, 46% had severely elevated anxiety, and 67% had elevated pregnancy-related anxiety, while social isolation strongly correlated with the likelihood of clinically significant depression or anxiety (Lebel et al., 2020; cited in Almeida et al., 2020).

There is some evidence that the COVID-19 pandemic has further exacerbated the pre-existing feelings of fear, stress, or loss of control and agency that women can experience during pregnancy, by adding the unknown factors about whether they or their newborns would test positive or be infected during labor, delivery, or the postpartum period, whether they would be permitted labor support, and whether having COVID-19 would further complicate their pregnancy through provider-induced preterm or cesarean delivery (Almeida et al., 2020). Shifting protocols in some hospitals began to allow labor support to women testing positive for SARS-CoV-2, as L&D nurse Lauren Hicks explained, women testing “positive are still being treated differently, but our protocols have recently improved. Now, COVID-19 positive mothers can have one companion with them, but the partner cannot leave the room during the whole hospital stay.” The rule insisting that the labor partner stay for days at a time discriminates against women whose partners work or care for small children at home without access to other caregivers. We have addressed the scarcity of care in disruptive times in the conclusion to our recent volume on sustainable birth across the globe (Gutschow et al., 2021).

### Shifting Practices: Mother-Newborn Separation and Transmission of SARS-CoV-2

Quite a few of our hospital-based providers reported that mothers testing positive for SARS-CoV-2 were separated from their newborns at birth, not allowed skin-to-skin contact, and discouraged from breastfeeding, based on the assumed possibility of mother-to-newborn COVID transmission. As MFM specialist Deena noted, “Some hospitals are sequestering newborns in the NICU if mothers are COVID-19 positive for up to 5 days, despite any evidence suggesting that this is beneficial.”

Obstetrician Michael White and L&D nurse Hicks both confirmed that their hospitals separated mothers who tested positive from their newborns. Yet Hicks noted some improvements in protocols: “Recently at my facility they have been allowed to breastfeed and have skin-to-skin contact with their newborns…I am so glad that now my facility is treating COVID-19 positive patients almost like any other patient.” Obstetrician Walters noted that his unit did not separate mothers and newborns, stating, “Babies need contact with their mom, and they need breast milk. We do allow breastfeeding and skin-to-skin, and advise hand washing and masks.”

For CNM Dinah Waranch, with her low-risk client base, the protocols about separation constituted one reason not to test asymptomatic mothers before birth:

Mothers are instructed/encouraged to test for COVID if they have symptoms or if they have a known exposure. We do not require prior to birth testing. This is partly because I do not believe my clientele would be happy to do that. It is also because I do not feel comfortable separating mother and baby after birth, which I regard as unnecessary and awful.

Returning to hospital births, unless mothers or newborns who tested positive were critically ill, they were usually sent home together within 2 days after birth even if they had been separated in the hospital. CNM Waranch responded to this paradox, stating: “No logic (to that), but then why expect logic from an illogical system?” Obstetrician Marco Giannotti added:

When the pandemic first started, I was a big proponent of keeping positive moms with their babies and breastfeeding. There just was not any data present indicating otherwise. I received a lot of pushback from our Neonatologists and Pediatricians. Shortly afterwards––the American Academy of Pediatrics confirmed that asymptomatic COVID positive moms should not be separated from their baby, and that breastfeeding should continue as normal.

CNM Kylea Liese confirmed that her hospital separated mothers and newborns in contradiction to AAP policy:

The rationale per peds [pediatrics] is “hospital policy” though they have acknowledged their own professional organization no longer supports this policy…the World Health Organization (WHO), Centers for Disease Control (CDC) and American Academy of Pediatrics (AAP) all recommend that mothers and babies stay together and breastfeed (if desired).

When the American Academy of Pediatrics (AAP, 2020) issued its first neonatal guidance on April 2, 2020, it recommended separating newborns from mothers who tested positive for SARS-CoV-2. Yet by September 9, 2020, the American Academy of Pediatrics (AAP, 2020) had issued new guidance recommending that “mothers and newborns may room-in according to usual center practices.” The later guidance urged doctors to discuss risks and benefits of rooming in with mothers and follow the mother’s choice, and also recommended delayed-cord clamping and skin-to- skin care for the mother and newborn, adding that mothers who tested positive should wear masks and practice handwashing prior to providing hands-on care for their newborns.

Nevertheless, the damage had been done. A CDC survey of 1,344 hospitals in the United States between July and August of 2020 (Perinne et al., 2020) confirmed that for mothers with suspected or confirmed COVID-19:

• Rooming in was discouraged in 38% and prohibited in 5% of hospitals
• Skin-to-skin care was discouraged in 14%, prevented in 6.5% of hospitals
• Skin-to-skin contact was only encouraged in 13% of hospitals
• Breastfeeding was discouraged in 20% of hospitals, but 17% of hospitals allowed feeding of expressed breastmilk

All of these policies were in direct contradiction to WHO, ACOG, and AAP guidance at the time, which strongly encouraged rooming-in, skin-to-skin contact, and breastfeeding for mothers with COVID-19, unless they were too ill to do so (Perinne et al., 2020). By August of 2020, the CDC had revised its guidance on rooming-in. The CDC recommended that mothers with suspected or confirmed SARS-CoV-2 infection discuss the risks and benefits of rooming in with their providers and that “healthcare providers respect maternal autonomy in the medical decision-making process.” As rationale, the CDC noted that;

Early and close contact between the mother and neonate has many well-established benefits. The ideal setting for care of a healthy, term newborn while in the hospital is in the mother’s room, commonly called “rooming in.” Current evidence suggests the risk of a neonate acquiring SARS-CoV-2 from its mother is low. Further, data suggests that there is no difference in risk of SARS-CoV-2 infection to the neonate whether a neonate is cared for in a separate room or remains in the mother’s room [Centers for Disease Control and Prevention (CDC), 2020b].

The guidance declared that mothers who test positive for SARS-CoV-2 should *not be considered* at risk of infecting their newborns if 10 days have passed since symptoms first appeared, at least 24 h have passed without a fever, and any other symptoms have improved [Centers for Disease Control and Prevention (CDC), 2020b]. We emphasize that these guidelines underscore our earlier point that mothers who are asymptomatic but test positive for SARS-CoV-2 are not necessarily infectious, as viral particles can be detected for days and even weeks after initial infection (Kaufman and Puopolo, 2021).

By December of 2020, further analysis of newborns whose mothers tested positive for SARS-CoV-2 revealed a low perinatal transmission rate to newborns (Schwartz et al., 2020a; Schwartz et al., 2020b; Schwartz and De Luca 2021) and supported the updated AAP and CDC guidance (Ronchi et al., 2020). A multicenter study from Lombardy, Italy of 62 newborns whose mothers tested positive for SARS-CoV-2 and who roomed-in with their mothers found that none of the newborns tested positive at birth, and after additional PCR tests at 7 and 20 days after birth, only 1.6% of newborns tested positive for the virus. Notably, nearly all newborns were breastfed (75% exclusively) and the study included newborns as young as 34 weeks (Ronchi et al., 2020).

A study of 120 newborns whose mothers tested positive for SARS-CoV-2 at a single New York City hospital also found that none of the newborns tested positive at birth (Salvatore et al., 2020). While only 68% of the newborns were followed up with a PCR test at 5–7 days of life, all newborns tested at 5–7 days and again at 14 days after birth (96% and 88% of the newborns followed up) tested negative and all were asymptomatic. Further, 83% of newborns had roomed-in with their mothers and many were breastfed or fed breastmilk by bottle (Salvatore et al., 2020). These studies (Ronchi et al., 2020; Salvatore et al., 2020) are in alignment with an AAP COVID-19 case registry that tested nearly 4,000 newborns in 2020: while 60% of the newborns roomed in with mothers, less than 2% of the newborns tested positive for SARS-CoV-2 (Kaufman and Puopolo, 2021).

The evidence on routes of newborn transmission continues to evolve. A meta-analysis of 176 newborns who tested positive for the virus in 2020 found that half of all newborns developed COVID-19 symptoms, roughly half were asymptomatic, and environmental transmission seemed more likely (70%) than intrauterine or intrapartum transmission (combined, 30%) (Raschetti et al., 2020). While unusual, transplacental transmission of SARS-CoV-2 does occur (Schwartz et al., 2020a) and the virus has been found in breastmilk (Groß et al., 2020). More studies are needed to understand the severity of COVID-19 in relation to other newborn complications, as many of the newborns who tested positive for SARS-CoV-2 were also preterm (Raschetti et al., 2020; Ronchi et al., 2020). A systematic review comparing rates of infection in newborns delivered vaginally vs. by cesarean across the globe confirmed that infection with SARS-CoV-2 is uncommon. Further, rates of infection do not differ significantly when comparing vaginal and cesarean delivery, breastfeeding or bottle-feeding, and babies rooming in vs. those separated to nurseries (Walker et al., 2020). More research is needed on the routes of vertical transmission, and on how admission to a NICU influences postnatal transmission or severity of COVID-19 in newborns.

In order to understand why hospitals moved so quickly to isolate newborns from mothers, it is important to recall that many standard obstetric practices are not evidence-based, cause harm (Miller et al., 2016), and have been analyzed as rituals that enact core technocratic values and generate a sense of safety for providers (Davis-Floyd, 2003; Davis-Floyd, 2018). This enactment of the old/new ritual of separation represents a reversion to the technocratic control that still characterizes mainstream obstetrics (Gutschow et al., 2021).

### Community Birth During COVID-19

The exclusion of doulas and support people has influenced the rising demand for births at home and in freestanding birth centers. As community-based midwife Willoughby puts it: “We saw a huge jump to OOH (out-of-hospital) at first, I think, because people had already hired their doulas and didn't want to lose the support.”

While our earlier survey indicated a significant increase in demand for community births (Davis-Floyd et al., 2020), the evidence was more mixed by November of 2020, with some providers reporting an increase and others seeing none. Homebirth obstetrician Fishbein did continue to see increased demand for home births in Los Angeles, and CNM Dinah Waranch of Texas noted:

a definite greater interest in OOH births…my practice has always been pretty busy and at capacity, but at my state of life (64) I’m not about to hire more to increase the size of practice during COVID. There are lots of area OOH practices which are taking up the slack.

For some providers and their clients, the rise of interest in community births did not always translate to a successful homebirth for a variety of reasons, as doula Stevie Merino noted:

I think there is definitely an increase in inquiries for home birth midwives but not an actual increase in follow through …. Many potential clients and current clients have reached out *via* email, social media, phone, and my website to ask for advice on how to find OOH options…Unfortunately, however many are unable to because of how far along they are in their pregnancy, insurance, cost of OOH options, high risk status, living situations, etc. I try to support however I can but also am realistic about people’s access and the fact that less than 2% of people in the United States still give birth in homes.

CPM Shea Childs from Arkansas described how she adapted to the growing demand for home birth in her area by asking more pointed questions about families’ motivations, and by noting that:

All the midwives in the state have had more families interested in home birth, but in a normal year there are only 250 or so families choosing OOH in the entire state, with the licensed midwives anyway. It will be interesting to see the numbers for 2020.

Community-based CPM Marimikel Potter of Texas described her reasons for rejecting some would-be clients:

When COVID-19 first got started, I got a bunch of calls from women wanting a home birth just because they were afraid of hospital infection. I rejected all of them because it was clear to me that they weren’t actually committed to home birth, and that rarely works out well.

CPM Sarita Bennett agreed, stating:

We didn’t accept those last-minute transfers at the beginning of the pandemic because the reasons for transferring didn’t give us confidence that the families were committed to our model of care and out of hospital birth. I’ve had several midwife friends regret that they accepted those transfers because they wound up with labor dystocias and transports way too often.

Here Bennett speaks to the notion that if a childbearing woman truly feels safer in the hospital she should deliver there, and that an ideological commitment to home birth can promote a successful outcome at home. LM Jessica Willoughby added:

When people were terrified of COVID and wanted to now have an OOH birth with almost no understanding of the difference in models of care I was like, wait, you’re afraid of COVID but what about MRSA or c-diff? What about all the other major communicable diseases that live in the hospital that you were already planning to risk exposure to when you signed up for hospital birth?

CPM Vicki Penwell, who runs a midwifery school in Boise, Idaho, saw a notable increase in demand for community births: “All the midwives all over the country that I have been speaking with recently are somehow managing to cope with client volumes of around eight births per month—twice their normal load. They are really rising to the challenge!” Yet this increased demand can add significant risks to midwives as births begin to cluster and practitioner stress and exhaustion set in. This could become a quality of care and safety issue if the demand remains high for too long; it clearly indicates the need for more community-based midwives.

Doula Stevie Merino added: “There is also an increased risk for OOH midwives who are already extremely restricted and regulated in the United States while also not being supported by most insurance options or by fair Medi-Cal reimbursements.” We are curious to see whether or not the increase in demand for what Melissa Cheney (2011) has called “the systems-challenging praxis” of home birth will continue post-COVID and influence integration of care in the United States.

### The Home/Hospital Divide in United States Maternity Care

Early in the pandemic, to help meet the rising demand for home births in New York, where it is illegal for CPMs to practice, Governor Cuomo issued Executive order 202.11 allowing midwives licensed anywhere in the United States and Canada to practice in New York State (Davis-Floyd et al., 2020). According to Ida Darragh, CPM and Chair of the North American Registry of Midwives (NARM), some CPMs from other states were able to work in New York, while others who had been practicing illegally in NY but licensed in another state were now able to practice legally. The governor extended this order in September 2020 with Chapter 182 of the Laws of 2020, which permitted the State Education Department to renew limited or provisional permits for midwives licensed in other states to continue practicing in New York for another 12 months.

It remains to be seen if the example set by New York State of accepting midwives licensed elsewhere will be followed by other states and whether momentum will build for more uniform acceptance of CPMs across all states, including in the 14 holdout states where they are still not allowed to practice legally. Clearly most obstetricians remain prejudiced against home births, as doula Merino described:

Many of my clients or potential clients who have discussed [the option of community birth] with their care providers have been told outright that it is still safer to birth in hospitals and it is actually “dangerous” to birth at home. This is quite ridiculous obviously and frankly a shame that even in the face of a pandemic that some hospital-based providers still do not see OOH providers as capable or see birth beyond a medical experience.

Many of the obstetricians we surveyed flatly stated they would not support home births during the pandemic:

Leslie Cohan: Absolutely not. Too risky. Want neo available, just in case.

Melinda Yates (a pseudonym): No I do not, why when you can have the same experience in the hospital and in the event of an emergency have everything you need.

Roberta Krueger: Studies show hospitals are safer than home birth.

Marco Giannotti: While I of course respect any patient’s decision when it comes to where she decides to deliver, I do feel that from a medical perspective, a hospital is *always* the safest place to deliver. Even during a pandemic. Should fetal compromise occur, the need to bring the patient to a hospital for emergent delivery takes precious time away from being able to quickly rescue a child in distress. One bad outcome is not acceptable.

Obstetrician Marilyn Vanover described community births as “risky,” yet blamed her obstetric colleagues by noting that they refused to perform or had minimal training in VBACs, vaginal breech deliveries, and other evidence-based practices. MFM specialist Deena had a very different view:

I do support OOH among people who have a trained CPM/CNM with good connections to facilitate transfer to a higher-level facility in case of issue…I tend to support this option more for multiparous people as adverse outcomes (e.g., need for transfer to hospital, C/S, transfusion, higher-order lacerations, need for operative vaginal delivery) are lower in this group. However, in the midst of the pandemic, I think a well-counseled person—understanding the risks and benefits of home birth—with a good care team and easy ability to access higher order care would be great as a home birth!

Deena’s optimism was not shared by obstetrician Giannotti at first. Yet when we presented with evidence of CPM-attended community births showing intrapartum and neonatal mortality rates that compared favorably with the outcomes of low-risk hospital births, Giannotti agreed that community births can be safe. Obstetrician Walters responded in ways that clearly argued for maternal agency and autonomy:

It is something of a challenge to answer this question. It is not a medical question. It is a human rights issue…a pregnant woman has the inalienable right to determine where she will deliver. There are risks and benefits to whichever location she chooses. Nothing is perfect.

When COVID appeared to be a serious threat to all pregnant women, Walters at first thought he might seek training in home deliveries, as he recognized that the skillset for home birth attendance was quite different from his own. He later abandoned this plan when his experience showed him that COVID did not present as much danger as initially feared:

We see now that COVID is a minimal threat to pregnant women and newborns. So, women are not avoiding our hospital. It is pretty much the same pros vs. cons of hospital vs. home birth to be weighed by the individual mom. And then I support that decision. I offer my skills and knowledge to help her achieve her goals. But, the patient decides... [the woman] has the right to know the qualifications and training of the person who will deliver her child. There is a massive difference between an experienced home birth CNM and some other “licensed” midwives. I am not an expert in the various forms of licensing for midwifery. But the ones I have seen make simple mistakes, miss diagnoses, mismanage deliveries, etc., have consistently been non-CNMs.

These provider responses indicate that there is a long way to go in educating obstetricians about the substantial evidence that exists showing excellent outcomes for planned, CPM-attended community birth in the United States (Johnson and Daviss 2005; Armstrong, 2010; Stapleton et al., 2013; Cheyney et al., 2014; Scarf et al., 2018).

### Home-to-Hospital Transfer in the Time of COVID-19

When we asked our providers if home birth transfer guidelines were being followed during home-to-hospital transport, CNM Waranch said that she is aware of the homebirth consensus transfer guidelines^5^, but “It’s difficult to implement them fully because the hospital is really not interested in meeting and doing that, virus or no virus.” In contrast, CNM Diana Jolles noted, “We have good transfer policies, and I would like to believe we follow the guidelines—which I adore—we are home birth midwives at heart, working in a large FQHC (Federally Qualified Health Center).”

CPM Debbie Query reported two hospital transfers: “One was quite smooth as I was able to transfer the charts as well as speak to the hospital staff. One was not as smooth according to the client, and (we both) feel that was predominately because I was not allowed to be there.” CPM Shea Childs saw no increase in transfers in her practice, but:

I think the level of stress has risen for everyone in the society. We have a mother/baby friendly hospital we transfer to and the care remains consistently positive. They are allowing one person to attend those laboring and a few are allowing a doula as well with some guidelines, like having preapproval from OB, that make the midwife going in with transfers a thing of the past sadly.

Homebirth obstetrician Stuart Fischbein also deeply disapproved of not being able to accompany his transporting clients:

Transfers are awful now! My experience is less integration. We as practitioners cannot accompany our clients in transport. It feels like forced abandonment. When we need to transport, we have to consider which facility will allow the father in the delivery or operating room. Which facility may allow the doula in….Many do not allow doulas. Many separate the mother from the partner, and some are not letting the father of the baby or partner into the operating room. When did fathers become non-essential personnel?

When Doula Merino had to switch to virtual labor support during transport to a hospital, she found:

that the cascade of interventions seems to increase—I can’t say for fact that it is related to the limited allowance of support persons but it has definitely felt that way…[preventing doulas seems] ridiculous and inefficient [because] the risk also seems the same since the laboring person was with their doula/midwife/or whoever else was present at home/birthing center with them prior to transfer.

Other providers also noted the increase in interventions when doulas were not present. CPM Sarita Bennett noted that one hospital in her region is off limits to her practice because it “won’t take transfers unless the person has been seen prenatally by one of the OBs on staff.” She describes how she helps her clients cope with transfer, virtually:

One client that transferred had to finally restrict entry to all those multiple pediatric residents trying to talk her into the Vitamin K and Hepatitis B injections that she had already declined. I could not physically accompany her but did the transfer by phone then stayed available by phone to the family to help them with information to advocate for themselves (like reminding her that she didn’t have to put up with all those pediatric residents).

LM Jessica Willoughby appreciates the value of the fact that she and her midwifery colleagues are now allowed back. She says that, in addition to the benefits to her clients of her hospital accompaniment:

I think that our presence at the hospital has helped with our reputation. We aren’t just dumping our patients on the hospital, we are going and helping facilitate communication between the hospital and the patient. I think that the providers at the hospital appreciate that. They see that we weren’t fueling this *United States vs. THEM* mentality. Listen hospital friend, we are all on the same spectrum here just different sides.

## Summary: Lessons Learned by Providers

Our respondents summarized the most significant lessons they learned in shifting their practices around COVID-19 as follows:

*Obstetrician Marco Giannotti:* The biggest lesson that we have learned (which is really an affirmation) is that patients need to be able to see their caregivers even when there is a pandemic.

*Obstetrician Jeffery Wright*: We are very thankful that reproductive age women are mostly unaffected by COVID. We were initially worried that it would be worse than H1N1. And we are even more grateful that newborns appear to be almost fully unaffected.

*Obstetrician Michael White*: For me the most significant lesson is the power and need for family support as we see the “social distancing” and elimination of the family’s involvement throughout a pregnancy.

*CNM Diana Jolles:* Big groups of people and organizations CAN work together quickly and effectively in the interest of public health…[Also] there are a lot of care practices pushed on midwives and communities that aren’t evidence based…

*CNM Dinah Waranch:* 1) midwife and client can listen to each other even when they have differing attitudes to the virus. This is heartwarming. and 2) I’d say we are at a point in my practice where we have our COVID system in place…It has been a gradual process to get clarity on the…guidelines and putting them into practice.... a constant state of refining.

*LM Jessica Willoughby:* We do not tolerate scientific ignorance in the birth center. You must wear a mask. Period.

*CNM Jenny Bagg:* For me personally, I learned that you can only trust yourself and what you are doing to protect yourself. You cannot rely on others to do the right thing.

*CPM Shea Childs:* Unfortunately, the takeaway is that folks are reluctant to take it seriously. Even though it is shared at beginning of care, many of the families seem to be shocked when I have had to relay that I have been exposed by a close contact, that I will not be seeing them for 2 weeks because of it and that if they go into labor, my back-up will be coming…Masking in an N95 is now second nature to me and that is the main change.

*Doula Stevie Merino*: Allowing some grace and patience with myself and others. I have also been more intentional about conversations with potential clients about the risks, my own work during this time, and the best practice protocols that I am practicing now. After being on call for months at a time, I finally learned that scheduled time off is important for my own overall health and wellbeing and that of my child.

*CPM and DO Sarita Bennett*: I believe that the lesson we should be learning is that large volume, facility birth is not sustainable and that small, community-based midwifery centers are the answer for the vast majority of pregnant people.

We highlight these responses here to show that in highly disruptive times of pandemics, United States-based providers adapt in ways that help their clients and their practice, using the lessons learned from experience. Our data has indicated that as the evidence shifts, so does practice among maternity care providers. We believe that dialogue among all kinds of providers (midwives and obstetricians, nurses, and doctors, community-based and hospital-based) promotes evidence-based care (Gutschow et al., 2021). We shift back and forth between community-based and hospital-based providers in our analysis because we believe that lessons from both settings can help shift practices most efficiently in highly disruptive times.

## Study Limitations and Strengths

There are several limitations to this study. It is based on a snowball sample of 28 survey responses and does not presume to speak for all United States maternity care practitioners. It is not geographically representative of all United States regions, although it is slightly skewed toward the urban and coastal United States. It does not reflect the racial, ethnic, and income diversity across the United States population. Only a few of our responses were from providers of color. Yet three-fourths of our respondents were female, who remain a minoritized community among United States-based physicians.^6^ Our survey represents a snapshot of time, of birth spaces, and of providers across the United States. Finally, it reflects the shifting guidance on COVID-19 that was not applied uniformly in all hospitals or by all providers.

The strengths of our study are that it illustrates in depth how some providers responded to a landscape in which much was shifting: evidence, client’s needs, as well as protocols or guidance from ACOG, AAP, WHO and other institutions. Our responses reached saturation, as later responses echoed earlier ones. Our study shows a variety of protocols among a range of providers who practice in different birth settings across the United States––home, birth center, and hospital. Finally, it describes changes in provider attitudes, experiences, and practices in their own words in response to the rapidly changing landscape of maternity care during the COVID-19 pandemic.

## Conclusion: Generating Integrated and Sustainable Maternity Care in Disruptive Times

The COVID-19 crisis represents a disruption or obstacle that is also an opportunity. It reveals the fractures in our current maternity care that might enable us to build a more sustainable and safer system of maternity care in which women can choose among multiple birth sites and multiple types of providers. We urge providers and policy makers to use these disruptive times to apply the lessons learned and work toward a leaner, more cost-effective, and *decentralized* maternity care system that integrates midwives with obstetricians and community birth providers with hospitals, while working to dismantle the systemic racism and provider bias that prevent high quality care for all (Gutschow et al., 2021; Daviss and Davis-Floyd, 2021).

There is ample evidence across the globe of sustainable models of birth that privilege midwifery models of care and provide high quality, high touch, low cost, and low-tech care (Davis-Floyd et al., 2009; Gutschow et al., 2021; Daviss and Davis-Floyd, 2021). We emphasize the teaching and transmission of midwifery skills and the midwifery model of care, which can be applied in home and hospital settings during chaotic times as well as more stable periods (Gutschow et al., 2021).

It is our hope that the fragmented maternity care system in the United States will become more integrated, by recognizing hospital- and community-based midwives and doulas as full participants in the care of mothers and newborns. In equalizing access to doulas, home birth, and freestanding birth centers through coordinated insurance schemes and subsidies, we may begin to improve health equity outcomes for minoritized populations in the United States and to de-racialize maternity care more broadly (Profit et al., 2020). We hope that community midwives can seize this pandemic moment to raise national awareness of their value, while obstetricians become more aware and accepting of the high value and cost-savings of midwifery care and community births (Daviss et al., 2021; Gutschow et al., 2021). Finally, we believe it critical that doulas be accepted as *essential* care providers, given the longstanding evidence that continuous doula support in labor reduces interventions and improves maternal and neonatal outcomes.

We hope that our maternity care system will restore humanistic strides made in facilitating normal physiologic birth and in enhancing maternal and newborn health. We hope that providers will work more collaboratively, with obstetricians recognizing midwives as colleagues rather than subordinates and doulas as essential, rather than non-essential, personnel. Finally, we believe that community midwives in the United States can achieve autonomous practice without restrictive state regulations, and thereby be empowered to practice and promote the midwifery model of care. In this way, they can continue to flexibly adapt to the next disruptions or crises that our society may face as recognized frontline providers—most especially when hospitals are overwhelmed. We hope that providers across the United States will seize the transformational moment of COVID-19 to transform the United States maternity care system to be more sustainable and more resilient in the face of future pandemics and disasters.

## Data Availability

The datasets presented in this article are not readily available because this dataset contains actual names of survey respondents and therefore should not be shared. Requests to access the datasets should be directed to kim.gutschow@williams.edu.

## Footnotes

1 All providers who responded to our emailed questionnaire gave written consent for their statements to be used in this article and most providers chose to be identified, while those who wished were anonymized, given a pseudonym, and all identifying details removed. Those who requested us to do so reviewed a draft before submission. Given these precautions, the Williams College IRB Chair exempted this research from IRB review.

2 This literature repository can be found at: http://hopkinshumanitarianhealth.org/empower/advocacy/covid-19/covid-19-children-and-nutrition/.

3 Data on global cases and deaths is from the global map at the John Hopkins University Coronavirus Resource center, at https://coronavirus.jhu.edu/map.html.

4 On March 28, 2020, Governor Cuomo of New York issued Executive Order 202.12 that explicitly allowed, “Any article 28 facility (public hospitals and nursing homes) licensed by the state, shall, as a condition of licensure permit the attendance of one support person who does not have a fever at the time of labor/delivery to be present for a patient who is giving birth.” (State of New York Executive Chamber, 2020).

5 These guidelines can be found at Best Practice Guidelines: Transfer from Planned Home Birth to Hospital|Home Birth Summit.

6 According to the Kaiser Family Foundation, while women have outnumbered men in entering medical school since 2010, male doctors (64% of all doctors) still outnumber female doctors (36% of all doctors) in the United States today.

## Appendix: Provider Questionnaire

### Changes in Practice in Response to the Pandemic

Where do you attend births? Home, freestanding birth center, in-hospital birth center, hospital maternity ward? In what city or state?
What are the major changes in your practice or protocols in response to COVID-19?
Do you use telehealth? If so, how do you use it and how is that working for you?
Do you make your clients/patients get tested for COVID pre-birth? Why or why not?
Do you and your staff (if any), get tested regularly for COVID?
Have any of your colleagues died from COVID exposure in your facility or practice?

### Attitudes towards COVID-19 among providers, pregnant people

What are the main fears that pregnant women have about COVID-19 during pregnancy, birth, and post-partum?
Are your staff afraid of contracting COVID-19? If so, how are their fears expressed?
How are COVID-19 positive women being treated in your facility/practice?
Are they allowed a labor companion, skin-to-skin contact immediately after birth, and to breastfeed? If not, why not—what is the rationale?
Do you perceive any racial bias in the treatment of COVID+ women? Or of any birthing people in your practice?

### Support People

Are doulas or support persons still being excluded from labor or birthing rooms or are they allowed? If so, one or the other, or both?
Is that support person allowed to stay post-partum and if so, for how long?

### OOH Births

Have you seen a continuing rise in demand for OOH (out-of-hospital) births, and if so, how is this rise being navigated in your facility, practice, or community?
Do you find that women choosing OOH birth simply due to fear of hospital contagion or of losing their chosen support people birth successfully at home or in a birth center, or end up transferring to hospital because that is where they feel safest?

### Transfers to Hospital

Have transfers between home to hospital increased or decreased in your estimation?
Are the transfers proceeding smoothly and are they following the “Best Practice Guidelines: Transfer from Planned Homebirth to Hospital” created in 2013 by the US Homebirth Consensus Summit?

### Other Issues

What are most significant lessons that you and your staff have learned from the pandemic thus far?
Are there other major ways in which your practice and protocols of maternity care have shifted in response to COVID-19 that you would like to discuss?
If we quote you in our article, do you prefer that we use your real name, or a pseudonym?
